# Supplementary material for: Global genetic differentiation of complex traits shaped by natural selection in humans
Source: Nat Commun. 2018 May 14;9:1865. doi: 10.1038/s41467-018-04191-y (PMC5951811; doi:10.1038/s41467-018-04191-y)
Supplement: Supplementary file 1 — Supplementary Information [file 41467_2018_4191_MOESM1_ESM.pdf]

Global genetic differentiation of complex traits shaped by natural selection in humans

Guo *et al.*

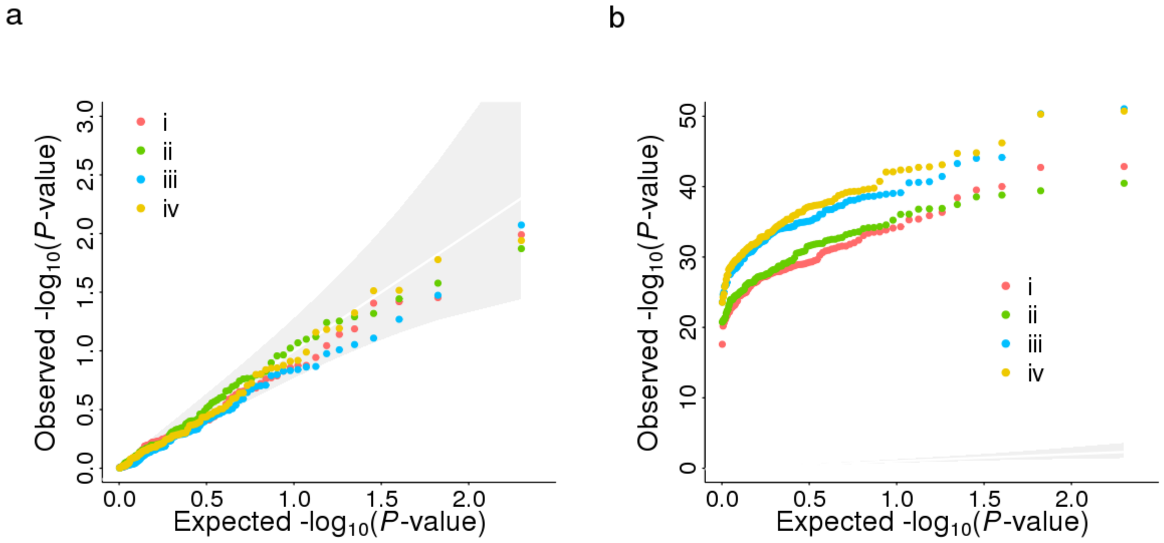

Supplementary Figure 1 Quantile-quantile plots of *P*-values generated from F_{ST} enrichment tests for the 100 simulated traits. The traits were simulated under 4 different settings (i-iv; Supplementary Table 3) under genetic drift (panel a) and selection (panel b) respectively. a. QQ plots of the test statistics under the null model (genetic drift). b. Power comparison under the alternative model (selection). The shaded area in panel (a) represents the 95% confidence interval (CI) of the expected *P*-values.

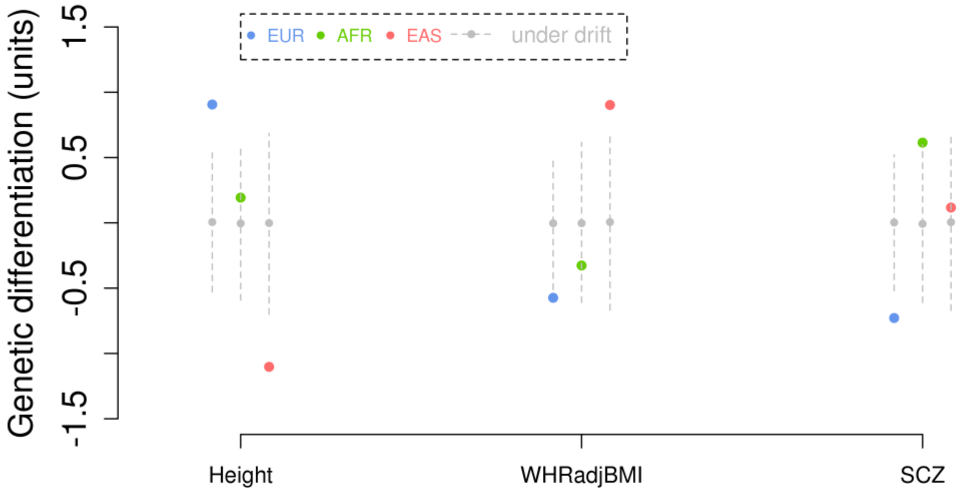

Supplementary Figure 2 Direction of genetic differentiation using the associated SNPs for height, WHRadjBMI and SCZ in the GERA populations. The colored dot represents the estimated deviation (in s.d. units) of the mean PRS based on the trait-associated SNPs clumped at $P < 5 \times 10^{-6}$ of a population from the overall mean across populations. The dot in gray represents the mean of mean PRS values of 10,000 sets of control SNPs, with the gray dashed line indicating the 95% confidence interval of the distribution of mean PRS values. WHRadjBMI, waist-to-hip ratio adjusted by BMI; SCZ, schizophrenia. EUR, European; AFR, African; EAS, East Asian.

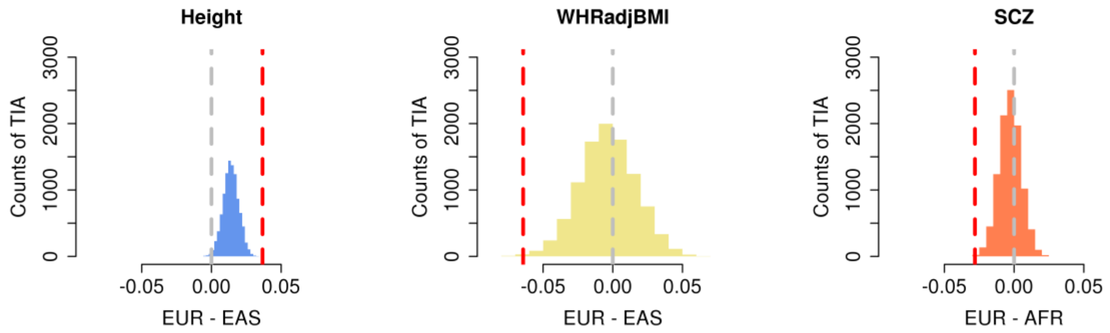

Supplementary Figure 3 Mean difference in frequencies of the trait-increasing alleles between two GERA populations for height, WHRadjBMI and SCZ. The red dashed line represents the mean difference in fTIA of the trait-associated SNPs clumped at $P < 5 \times 10^{-6}$. The histogram represents the distribution of the difference in fTIA for the control SNPs. The gray dashed line represents the expected difference in fTIA (i.e., 0) under genetic drift. WHRadjBMI, waist-to-hip ratio adjusted by BMI; SCZ, schizophrenia; EUR, European; AFR, African; EAS, East Asian.

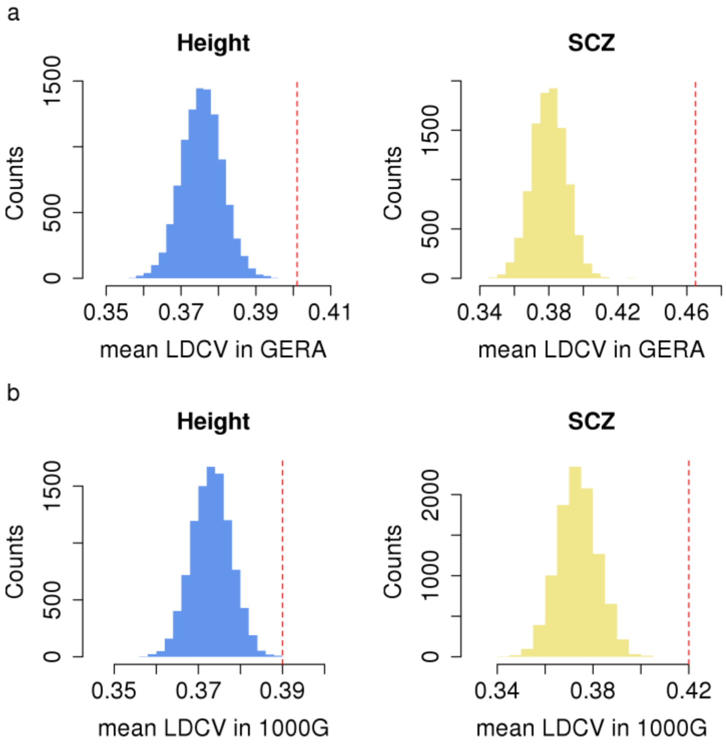

Supplementary Figure 4 Testing the mean LDCV of the associated SNPs (clumping $P < 5 \times 10^{-6}$) against the distribution based on the control SNPs for height and SCZ in GERA (a) and 1000G (b). SCZ, schizophrenia.

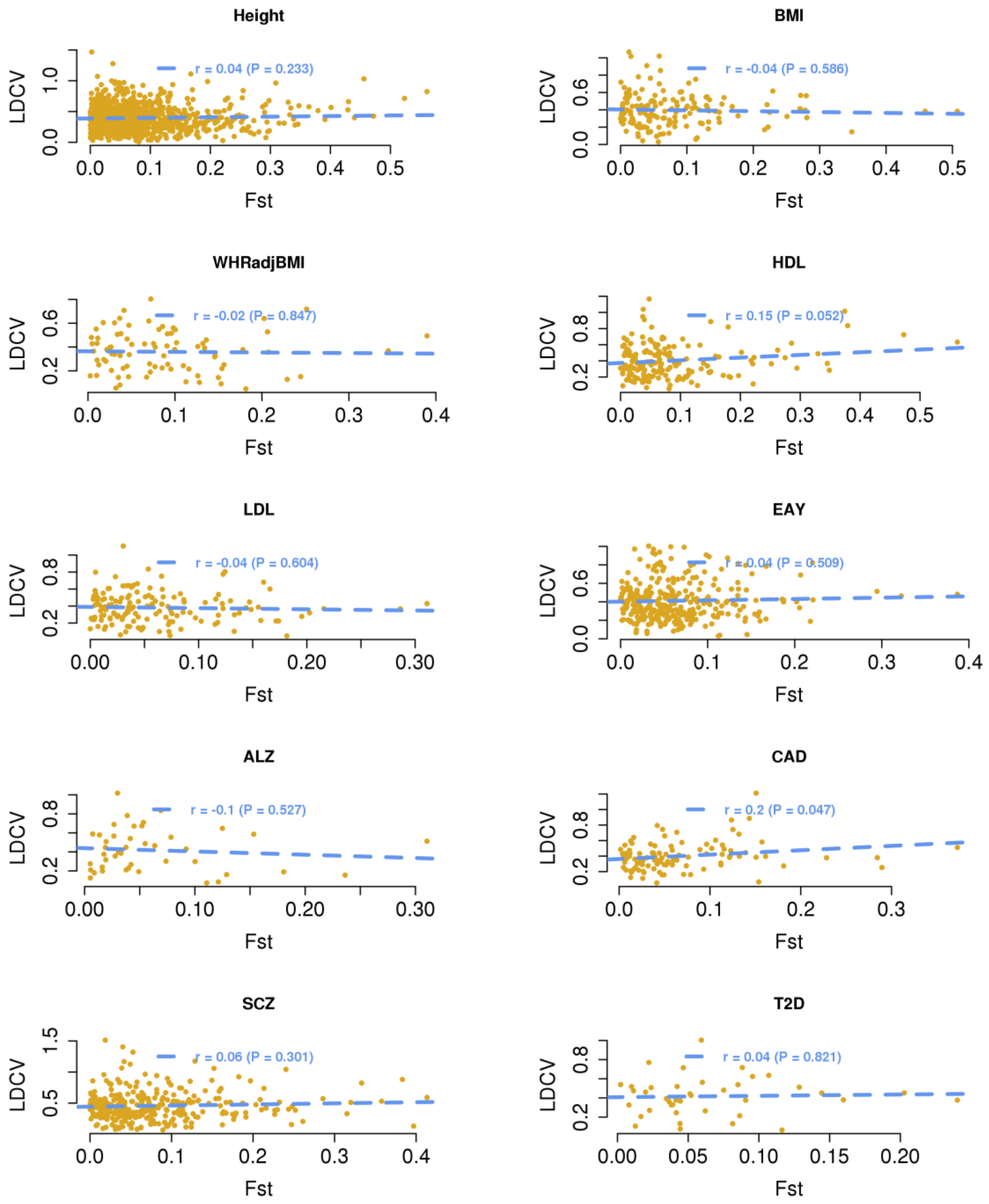

Supplementary Figure 5 Correlation between the F_{ST} (y-axis) and LDCV (x-axis) of the trait-associated loci (clumped at $P < 5 \times 10^{-6}$) for ten complex traits. r and P represent the estimates of Pearson correlation coefficient and the corresponding P-values, respectively.

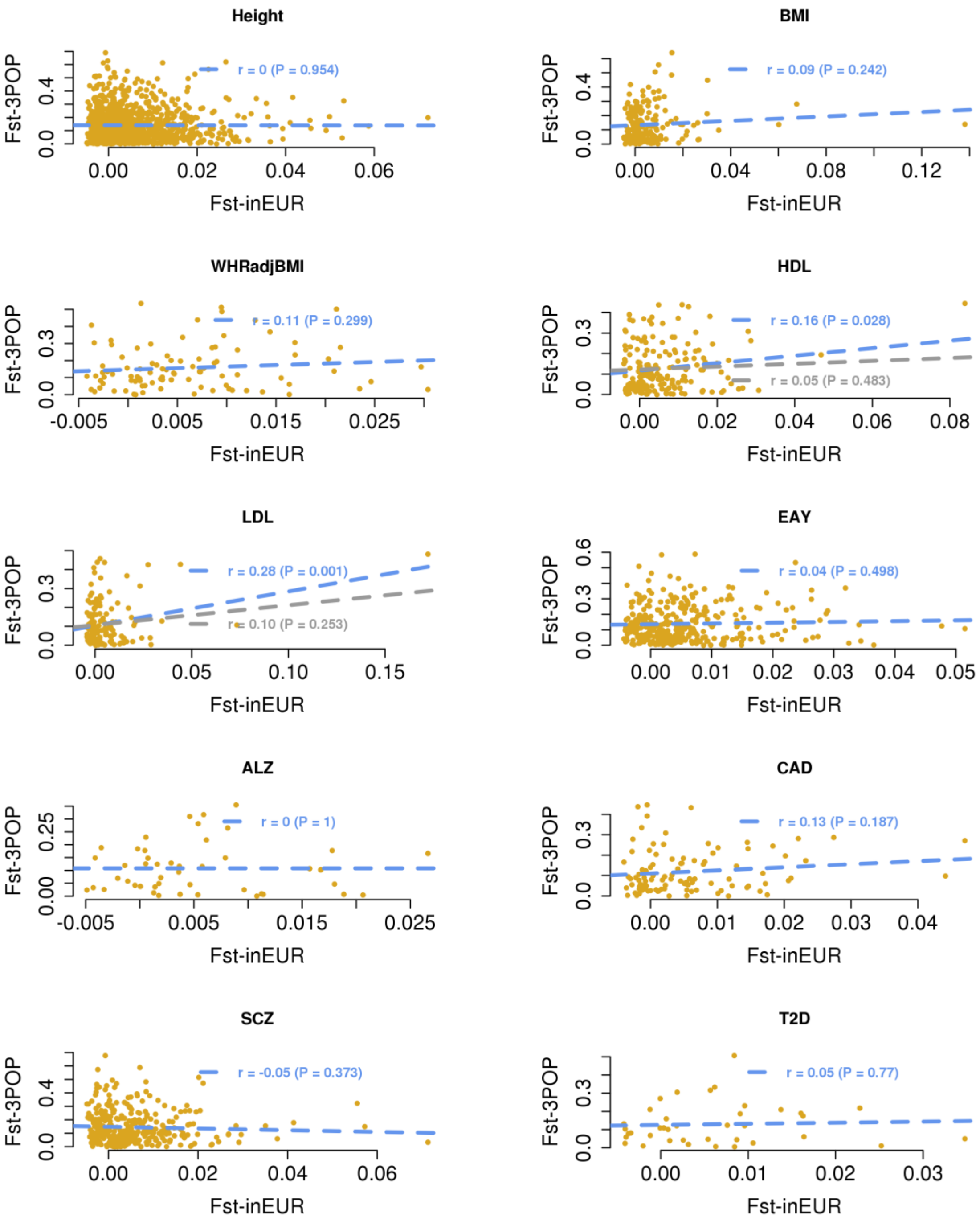

Supplementary Figure 6 Correlation between the worldwide F_{ST} (y-axis) and European F_{ST} (x-axis) of the trait-associated loci (clumped at $P < 5 \times 10^{-6}$) for ten complex traits. r and P represent the estimates of Pearson correlation coefficient and the corresponding P-values, respectively. The significant correlations for HDL and LDL (blue dash line) were driven by two outliers. The results without the outliers were presented in gray.

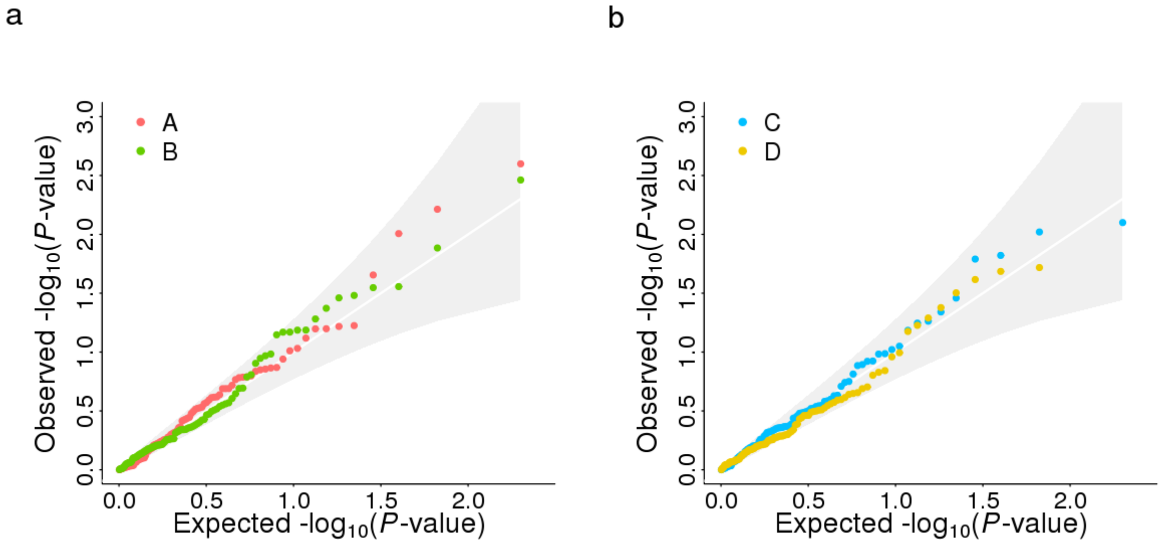

Supplementary Figure 7 Quantile-quantile plots of *P*-values generated from F_{ST} enrichment tests for two additional simulations: one based on causal variants with lower MAF (MAF < 0.1; panel a) and the other with lower heritability (h^2 = 0.2; panel b) (Supplementary Table 4) under genetic drift. We used a *P*-value threshold of 5×10^{-8} for the clumping in A and C and 5×10^{-6} in B and D. The shaded area represents the 95% CI of the expected *P*-values.

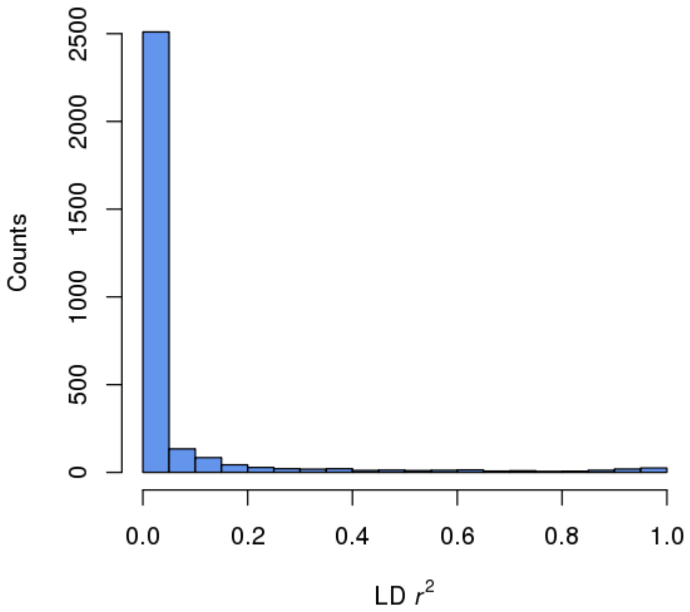

Supplementary Figure 8 Distribution of LD r^2 between the simulated multiple causal variants at each locus. In this simulation scenario, we sampled 1,000 causal variants at random across the genome with additional two causal variants from a 1-Mb flanking region of each of the primary causal variants (see Methods for more details of the simulation design). The LD r^2 values were computed in the 53,629 unrelated EUR in GERA.

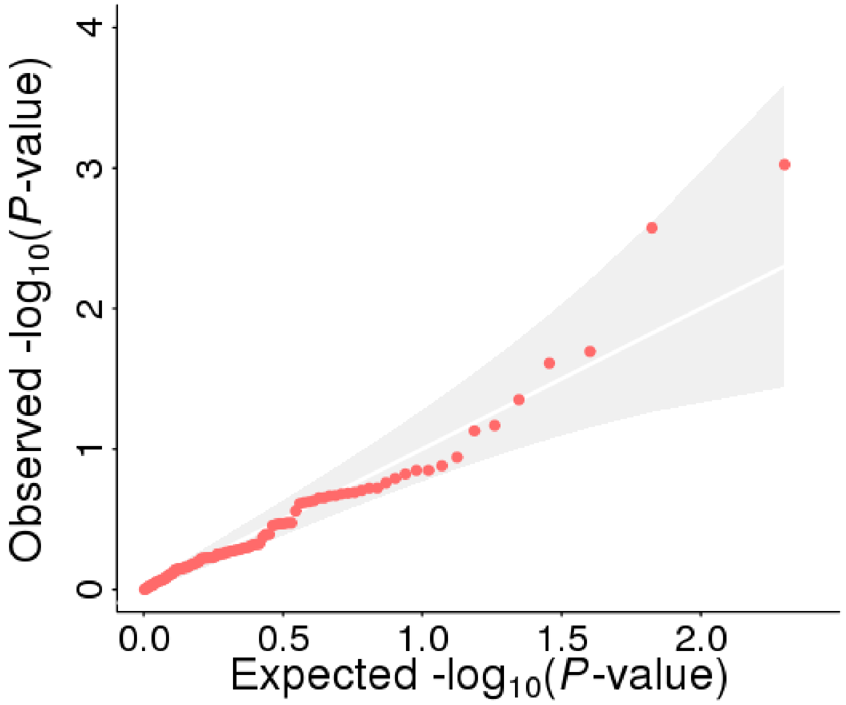

Supplementary Figure 9 Quantile-quantile plot of *P*-values from the F_{ST} enrichment analysis in the simulation with two additional causal variants in a 1-Mb flanking region of each primary causal variant (see Supplementary Fig. 8 for the distribution of LD between the causal variants at each locus and see Methods for more details of the simulation design). The shaded area represents the 95% CI of the expected *P*-values.

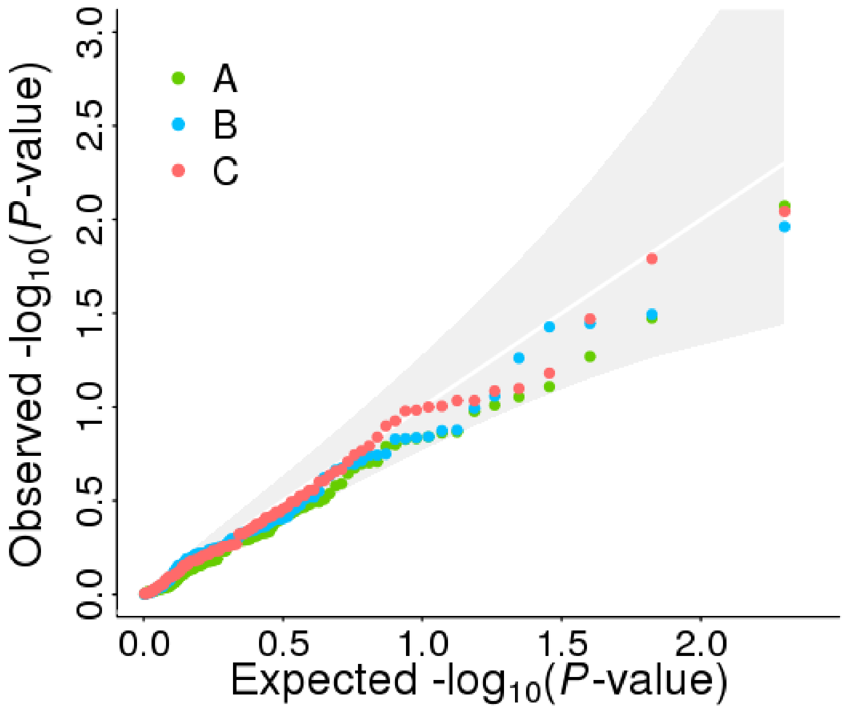

Supplementary Figure 10 Quantile-quantile plots of *P*-values from three different strategies of sampling control SNPs in the F_{ST} enrichment analysis of simulated traits (see Methods for more details of the simulation design). We matched the control SNPs with the trait-associated SNPs by A) both MAF and LD estimated in EUR (as with the strategy used in real data analysis), B) only MAF estimated in EUR, and C) both MAF and LD estimated in AFR. The shaded area represents the 95% CI of the expected *P*-values.

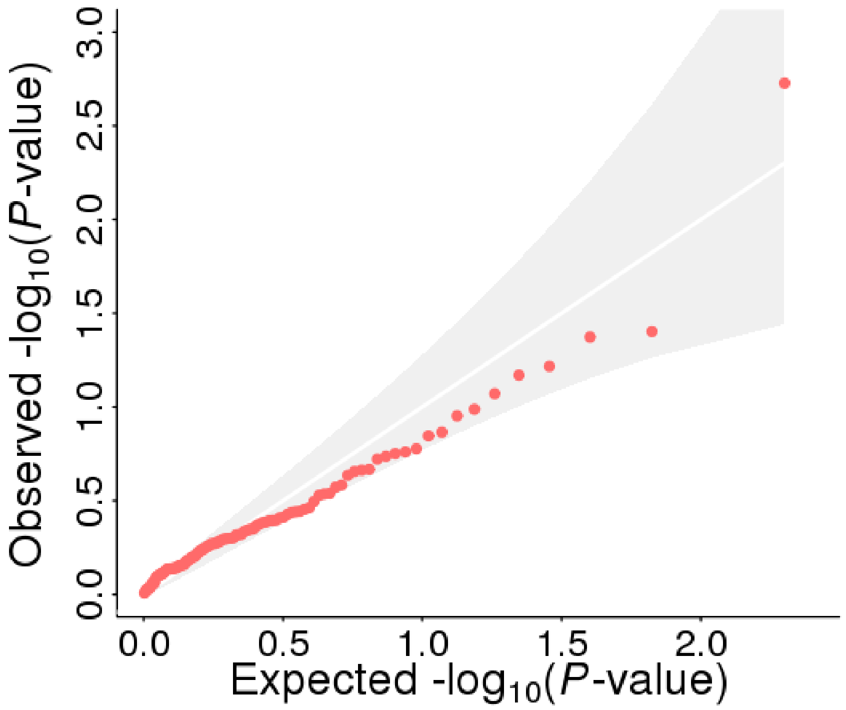

Supplementary Figure 11 Quantile-quantile plot of *P*-values generated from the F_{ST} enrichment analysis of simulated traits with causal variants being excluded from the analysis. The shaded area represents the 95% CI of the expected *P*-values.

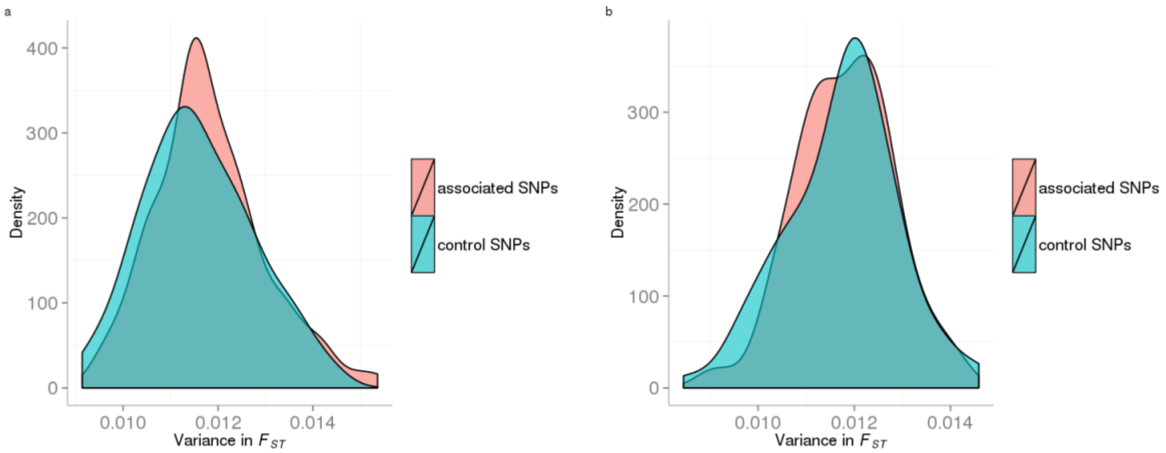

Supplementary Figure 12 Comparison of the distribution of the variance in F_{ST} of the trait-associated SNPs with that of a random set of control SNPs for 100 simulated traits. The traits were simulated independently based on 1,000 causal loci randomly sampled from GERA-EUR genotypes with a single variant (panel a) or three variants (panel b) at each of the causal loci (see Methods for details of the simulation). In panel a), the mean of the distribution is 0.0118 ($\mathrm{s.e.m.} = 0.00012$) for the trait-associated SNPs (pink) and 0.0116 ($\mathrm{s.e.m.} = 0.00012$) for the control SNPs (blue). The corresponding values in panel b) are 0.0118 ($\mathrm{s.e.m.} = 0.0001$) and 0.0118 ($\mathrm{s.e.m.} = 0.00012$). The mean of the distribution for the associated SNPs is not significantly different from that for the control SNPs ($P_{\mathrm{difference}} = 0.193$ in panel a and $P_{\mathrm{difference}} = 0.689$ in panel b).

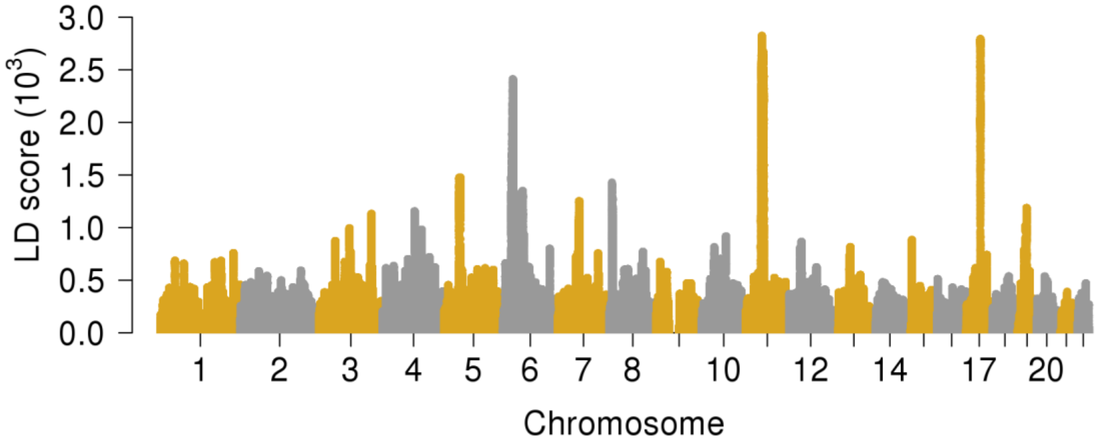

Supplementary Figure 13 LD scores of all SNPs on 22 autosomes in GERA-EUR.

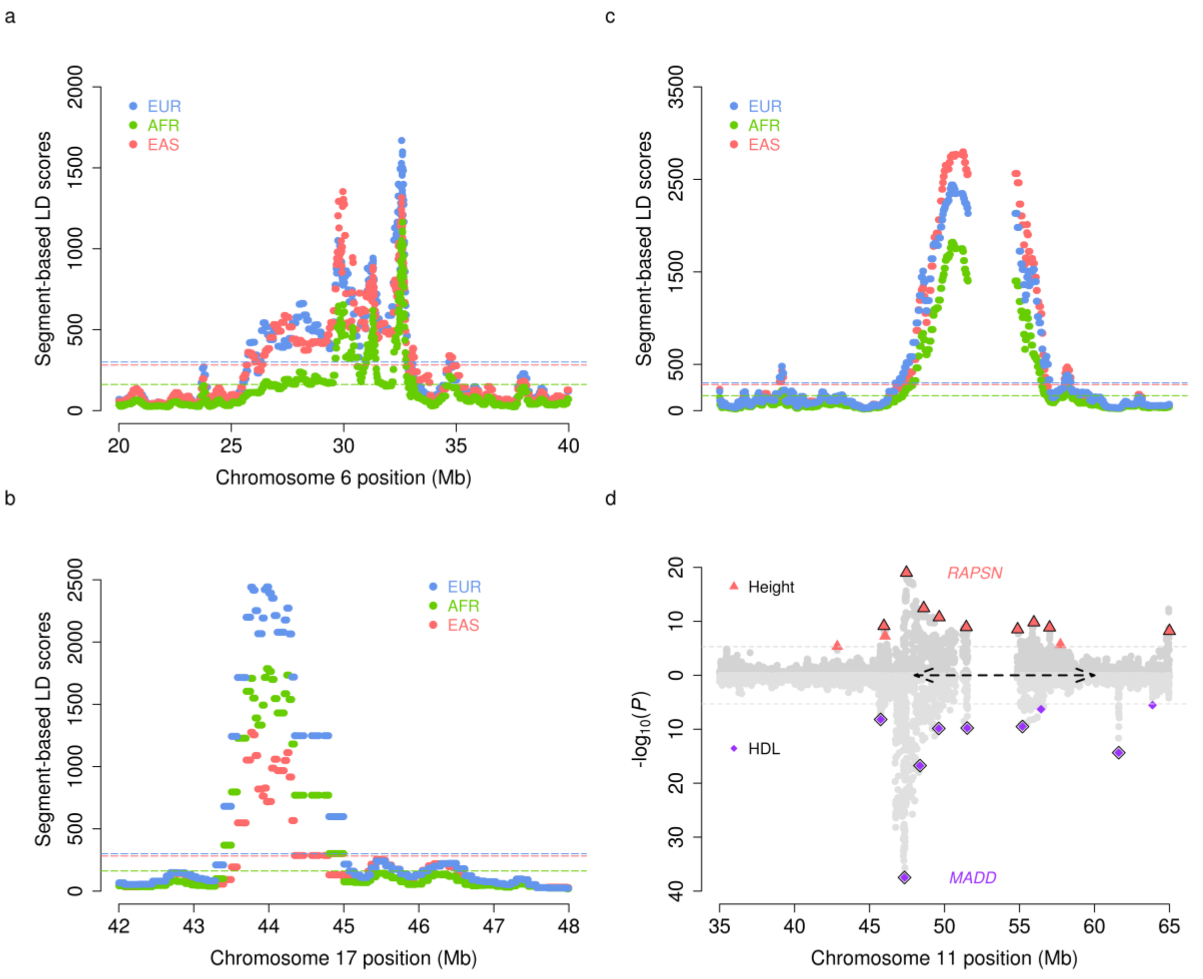

Supplementary Figure 14 Three regions with extremely large LD scores in the three populations. These regions are on chromosome 6 (panel a), 17 (panel b) and 11 (panel c and d). There are a number of SNPs in the chr11 region showing pleiotropic effects on height and HDL (panel d). In panels a, b and c, the 95th percentile of the segment-based LD score distribution for each population is represented by the long-dash lines. The clumping-selected ($P < 5 \times 10^{-6}$) loci for height and HDL-cholesterol are labelled as triangles (red) and diamonds (purple) respectively with the GWAS significant SNPs (clumping $P < 5 \times 10^{-8}$) indicated with black border (panel d). Panels c and d share the same x-axis. In panel d, the gray dashed lines indicate the clumping P-value threshold (5×10^{-6}) and the dashed line with arrows indicates the region with an strong enrichment of OR genes (186 ORs/308 genes). *RAPSN* and *MADD* are the two genes containing the leading SNPs in this region for height (rs7126210; hg38 chr11:47,438,755; $P = 9.9 \times 10^{-20}$) and HDL-cholesterol (rs3847502; hg38 chr11:47,312,134; $P = 3.31 \times 10^{-38}$) respectively, both of which locate in the coding region (hg38 chr11:47,437,975-47,448,964 for *RAPSN* and hg38 chr11:47,273,915-47,329,150 for *MADD*)[1]. EUR, Europeans; AFR, Africans; EAS, East Asians.

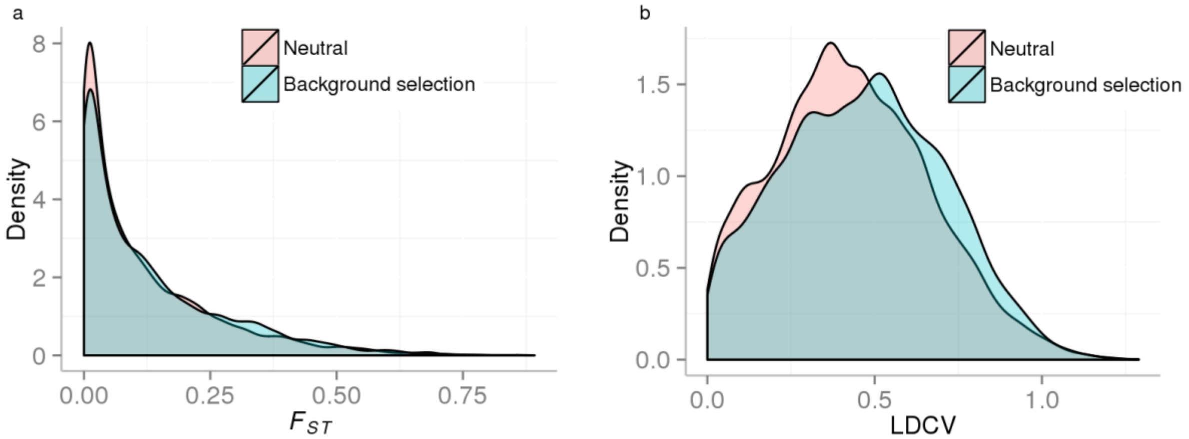

Supplementary Figure 15 Comparison of the distributions of F_{ST} and LDCV between the neutral variants and those under background selection. Shown are the results from one replicate of the forward simulation using SLiM[45] (see Methods for details of the simulation). In brief, we conducted a forward simulation to mimic the "Out of Africa" event based on a commonly used demographic model[63]. We simulated two independent 10-Mb segments, one with 5% of the mutations being deleterious for fitness and 95% being neutral and the other with all the mutations being neutral. We simulated negative selection on the deleterious variants so that the neutral variants on segment #1 in LD with the deleterious variants were under background selection while all the variants on segment #2 were unaffected. We repeated the simulation 30 times. Over 30 replicates, the average number of neutral variants was 16,860 (s.e.m. = 155), significantly larger than that of the variants under background selection (mean = 12,808 and s.e.m. = 109). The mean of mean F_{ST} values for the neutral variants across 30 replicates was 0.117 (s.e.m. = 0.002), which was significantly lower than that of the MAF- and LD-matched variants under background selection (mean = 0.137 and s.e.m. = 0.002) (panel a). The corresponding value for LDCV was 0.411 (s.e.m = 0.003), also significantly lower than that under background selection (mean = 0.44 and s.e.m = 0.004) (panel b).

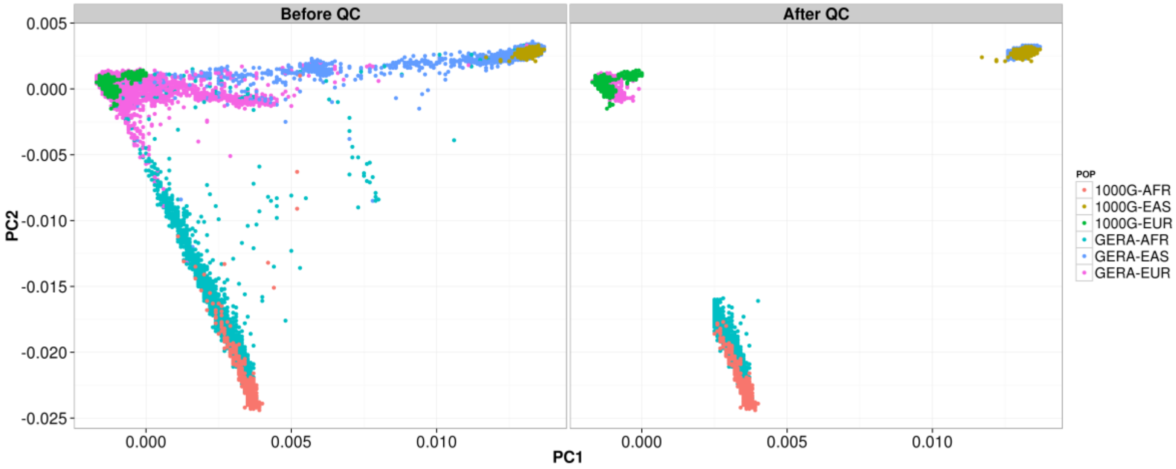

Supplementary Figure 16 Quality control (QC) of the GERA samples based on the principal component analysis. EUR, Europeans; AFR, Africans; EAS, East Asians.

Supplementary Table 1 Description of the GWAS summary statistics for 10 complex traits.

Traits	n (n_{case}/$n_{control}$)		#SNPs			Publications
			Before QC	Overlapped SNPs		
				GERA	1000G	
Height	253,288	-	2,550,858	1,857,980	2,012,883	Wood et al. 2014 Nature Genetics
BMI	322,154	-	2,554,637	1,862,451	2,017,892	Locke et al. 2015 Nature
WHRadjBMI	210,088	-	2,542,431	1,839,639	1,992,582	Shungin et al. 2015 Nature
HDL	188,577	-	2,447,441	1,812,965	1,957,333	GLGC 2013 Nature Genetics
LDL	188,577	-	2,437,751	1,809,875	1,954,298	GLGC 2013 Nature Genetics
EAY	405,072	-	8,146,840 (5,000*)	5,098,341 (3,064)	5,006,350 (2,940)	Okbay et al. 2016 Nature
AD	17,008	37,154	7,055,881	4,696,485	4,665,245	Lambert et al. 2013 Nature Genetics
CAD	60,801	123,504	9,455,778	5,207,439	5,023,631	Nikpey et al. 2015 Nautre Genetics
SCZ	36,989	113,075	9,444,230	5,138,061	5,008,754	PGC 2014 Nature
T2D	12,171	56,862	2,473,441	1,813,058	1,960,560	Morris et al 2012 Nature Genetics

BMI, body-mass-index; WHRadjBMI, waist-to-hip ratio adjusted by BMI; HDL, high-density lipoprotein cholesterol; LDL, low-density lipoprotein cholesterol; EAY, education attainment years; AD, Alzheimer's disease; CAD, coronary artery disease; SCZ, schezophrenia; T2D, type II diabetes; n, sample size; #SNPs, number of SNPs in the data. *5,000 top associated SNPs for EAY from the Social Science Genetic Association Consortium meta-analysis including the 23andMe samples (Okbay et al. 2016 Nature).

Supplementary Table 2 Sample sizes of GERA and 1000G populations.

	GERA			1000G	
	**Original**	**Unrelated**	**Final**	**Original**	**Unrelated**
**EUR**	60,586	53,629	1,099	503	494
**AFR**	3,826	1,099	1,099	661	591
**EAS**	5,188	3,365	1,099	504	491

Shown are the original sample sizes of the GERA and 1000G populations, the number of unrelated individuals (genetic relatedness < 0.05) after QC, and final sample sizes in GERA after down-sampling.

Supplementary Table 3 Four simulation scenarios under the null and alternative hypotheses including two *P*-value thresholds for clumping and two SNP pools to sample control SNPs for the F_{ST} enrichment test.

**Simulation scenario**	h^2	**#causal variants**	**Clumping**			**SNP Pool**
			***P*-value**	**LD r^2**	**Window (Kb)**	
i	0.5	1000	5×10^{-8}	0.01	1000	1000G
ii	0.5	1000	5×10^{-8}	0.01	1000	HapMap2
iii	0.5	1000	5×10^{-6}	0.01	1000	1000G
iv	0.5	1000	5×10^{-6}	0.01	1000	HapMap2

h^2, trait heritability.

Supplementary Table 4 Two additional simulation scenarios under genetic drift: 1) 1,000 random causal variants with 500 additional causal variants with MAF < 0.1 and 2) 1,000 random causal variants with lower heritability ($h^2 = 0.2$).

Simulation scenario	h^2	#causal variants		Clumping			SNP pool
		Random MAF	MAF < 0.1	*P*-value	LD r^2	Window (Kb)	
A	0.5	1000	500	5×10^{-8}	0.01	1000	1000G
B	0.5	1000	500	5×10^{-6}	0.01	1000	1000G
C	0.2	1000	-	5×10^{-8}	0.01	1000	1000G
D	0.2	1000	-	5×10^{-6}	0.01	1000	1000G

Supplementary Table 5 F_{ST} enrichment analysis with different strategies of sampling control SNPs. We matched the control SNPs with the trait-associated SNPs by I) only MAF estimated in 1000G-EUR and II) both MAF and LD estimated in 1000G-AFR.

**Trait**			**I**	**II**
	**#SNPs**	**mean F_{ST}**	***P*-value**	***P*-value**
Height	1,099	0.140	1.08×10^{-5}	8.81×10^{-6}
BMI	179	0.136	0.264	0.228
WHRadjBMI	92	0.158	3.9×10^{-3}	2.1×10^{-3}
HDL	181	0.128	0.721	0.639
LDL	139	0.115	0.360	0.469
EAY	328	0.139	0.015	9.39×10^{-4}
AD	46	0.108	0.530	0.949
CAD	102	0.119	0.961	0.738
SCZ	337	0.145	1.18×10^{-4}	1.97×10^{-5}
T2D	38	0.129	0.826	0.556

#SNPs, number of SNPs.

Supplementary Table 6 Quality control criteria of the genotyped and imputed data in GERA and ARIC.

**Data**	**QC**	**Exclusive criteria**	
		**GERA**	**ARIC**
**Genotyping**	Sample call rate	< 98%	< 98%
	SNP call rate	< 98%	< 98%
	MAF	< 0.01	< 0.01
	HWE *P*-value	$< 10^{-6}$	$< 10^{-3}$
**Imputed**	MAF	< 0.01	< 0.01
	HWE *P*-value	$< 10^{-6}$	$< 10^{-6}$
	INFO score	< 0.3	< 0.3

MAF, minor allele frequency; HWE, Hardy-Weinberg equilibrium

Supplementary Note: Acknowledgements

ARIC: The Atherosclerosis Risk in Communities Study is carried out as a collaborative study supported by National Heart, Lung, and Blood Institute contracts (HHSN268201100005C, HHSN268201100006C, HHSN268201100007C, HHSN268201100008C, HHSN268201100009C, HHSN268201100010C, HHSN268201100011C, and HHSN268201100012C), R01HL087641, R01HL59367 and R01HL086694; National HumanGenome Research Institute contract U01HG004402; and National Institutes of Health contract HHSN268200625226C. The authors thank the staff and participants of the ARIC study for their important contributions. Infrastructure was partly supported by Grant Number UL1RR025005, a component of the National Institutes of Health and NIH Roadmap for Medical Research.

GERA: The Genetic Epidemiology Research on Adult Health and Aging study was supported by grant RC2 AG036607 from the National Institutes of Health, grants from the Robert Wood Johnson Foundation, the Ellison Medical Foundation, the Wayne and Gladys Valley Foundation and Kaiser Permanente. The authors thank the Kaiser Permanente Medical Care Plan, Northern California Region (KPNC) members who have generously agreed to participate in the Kaiser Permanente Research Program on Genes, Environment and Health (RPGEH).

References

1. Kent, W. J. *et al.* The human genome browser at UCSC. *Genome Res.* **12,** 996–1006 (2002).
